# Age and Prostate-Specific Antigen Level Prior to Diagnosis Predict Risk of Death from Prostate Cancer

**DOI:** 10.3389/fonc.2016.00157

**Published:** 2016-06-28

**Authors:** F. Roy MacKintosh, Preston C. Sprenkle, Louise C. Walter, Lori Rawson, R. Jeffrey Karnes, Christopher H. Morrell, Michael W. Kattan, Cayce B. Nawaf, Thomas B. Neville

**Affiliations:** ^1^VA Sierra Nevada Health Care System, Reno, NV, USA; ^2^VA Connecticut Healthcare System, Yale School of Medicine, New Haven, CT, USA; ^3^Division of Geriatrics, San Francisco VA Medical Center, University of California San Francisco, San Francisco, CA, USA; ^4^Mayo Clinic, Rochester, MN, USA; ^5^Loyola University Maryland, Baltimore, MD, USA; ^6^Department of Quantitative Health Sciences, Cleveland Clinic, Cleveland, OH, USA; ^7^Department of Urology, Yale School of Medicine, New Haven, CT, USA; ^8^Soar BioDynamics, Inc., Incline Village, NV, USA

**Keywords:** prostate cancer, prostate-specific antigen, life expectancy, older men, death risk

## Abstract

A single early prostate-specific antigen (PSA) level has been correlated with a higher likelihood of prostate cancer diagnosis and death in younger men. PSA testing in older men has been considered of limited utility. We evaluated prostate cancer death in relation to age and PSA level immediately prior to prostate cancer diagnosis. Using the Veterans Affairs database, we identified 230,081 men aged 50–89 years diagnosed with prostate cancer and at least one prior PSA test between 1999 and 2009. Prostate cancer-specific death over time was calculated for patients stratified by age group (e.g., 50–59 years, through 80–89 years) and PSA range at diagnosis (10 ranges) using Kaplan–Meier methods. Risk of 10-year prostate cancer mortality across age and PSA was compared using log-rank tests with a Bonferroni adjustment for multiple testing. 10.5% of men diagnosed with prostate cancer died of cancer during the 10-year study period (mean follow-up = 3.7 years). Higher PSA values prior to diagnosis predict a higher risk of death in all age groups (*p* < 0.0001). Within the same PSA range, older age groups are at increased risk for death from prostate cancer (*p* < 0.0001). For PSA of 7–10 ng/mL, cancer-specific death, 10 years after diagnosis, increased from 7% for age 50–59 years to 51% for age 80–89 years. Men older than 70 years are more likely to die of prostate cancer at any PSA level than younger men, suggesting prostate cancer remains a significant problem among older men (even those aged 80+) and deserves additional study.

## Introduction

Each year, prostate cancer claims the lives of nearly 307,000 men worldwide and nearly 30,000 men in the United States (1). The prostate cancer death rate in the United States has been nearly halved since the beginning of prostate-specific antigen (PSA)-based screening over 25 years ago (2). However, the benefits of PSA screening, and hence the PSA test itself, have been debated recently after the European Randomized Study of Screening for Prostate Cancer (ERSPC) (3) and the Prostate, Lung, Colorectal, and Ovarian Cancer Screening Trial (PLCO) in the United States (4) revealed conflicting evidence about the benefit of PSA screening.

The controversy about PSA screening implicitly questions the importance of diagnosing and treating prostate cancer. Understanding the magnitude of prostate cancer death, and which men are at higher risk of death, may identify those who could benefit from treatment. PSA level and older age prior to biopsy have independently been identified as risk factors for prostate cancer diagnosis, but their relationship with prostate cancer death has not been adequately addressed in the literature.

Stephenson et al., in a multi-institutional study, demonstrated that a higher PSA prior to surgical treatment leads to a higher risk of cancer-related death at 10 years, with the risk of death from prostate cancer doubling if the pretreatment PSA increases from <4 ng/mL to 10.1–20 ng/mL (5). However, this study did not consider age and was limited to only patients who underwent surgery, which is typically a healthy subset of men. A baseline or early PSA test result in younger men has been demonstrated in many studies to correlate with the likelihood of prostate cancer diagnosis (6–10) during their lifetime, and even likelihood of developing metastases (8) and prostate cancer-specific death (8, 10–12).

These baseline and early PSA studies did not address whether patients should have a biopsy, though several risk calculators attempt to inform that decision. The ERSPC risk calculator estimates the probability a biopsy will find prostate cancer as a function of prior PSA and other variables but not age (13–15). The Prostate Cancer Prevention Trial (PCPT) risk calculator, on the other hand, estimates the probability of detecting prostate cancer with a biopsy as a function of age, prior PSA, and other variables – with the probability of a cancer diagnosis increasing as PSA and age prior to biopsy rise (16–19).

The increasing risk of a cancer diagnosis with age is likely to be offset, to some extent, by a reduced life expectancy for older men who may die of other causes before prostate cancer. Furthermore, over the last 50 years, there has been a steady decline in cardiac-related death, the most common killer of Americans, and life expectancy continues to increase. The age-adjusted death rate from cardiovascular disease decreased almost 50% between 1963 and 2010, even between 1990 and 2010, a 20% decrease was noted (20). With an increasingly healthy and older population, prostate cancer may become a more relevant cause of death. None of the published literature has estimated the risk of prostate cancer death for men diagnosed with prostate cancer as a function of age and PSA prior to biopsy for any population, whether small or large. An understanding of the risk of cancer death, not just the likelihood of cancer detection, could inform the decision for older men whether to proceed to a biopsy.

In order to more clearly understand the magnitude of prostate cancer death in an aging population, we sought to evaluate the relationship between age and PSA prior to biopsy with the risk of subsequent prostate cancer death in a large national population of men diagnosed with prostate cancer.

## Materials and Methods

### Participants and Data Sets

This is a cohort study of 230,081 men aged 50–89 years diagnosed with prostate cancer in the Veterans Administration (VA) between 1999 and 2009. The goal of the study is to determine the risk of prostate cancer death during this 10-year time period according to age and PSA level at diagnosis. Data for this study were compiled from all VA facilities across the United States, available in the corporate data warehouse (CDW). Men diagnosed with prostate cancer were identified by (1) a diagnosis code in the patient record or (2) a surgery code in the patient record for men with no diagnosis code. Diagnosis date was determined to be the earliest date of diagnosis found in one of four sources where the International Classification of Diseases 9th edition (ICD-9) code of *185* appeared: the VA Central Cancer Registry data file; the CDW outpatient table; the CDW inpatient table; or the Surgical Quality Data Use Group (SQDUG) data file. PSA test values were identified by the presence of a Logical Observation Identifiers Names and Codes (LOINC) code *2857-1* in the CDW Patient Laboratory Chemistry (LabChem) table. When available, numeric values were used for PSA test results. In the absence of a numeric value, the text was parsed and analyzed to identify a PSA test result. Duplicate PSA values were purged. The PSA test result immediately prior to diagnosis was used as the PSA value at diagnosis. The VA vital status mini table of the CDW provided patient demographics, including date of birth, which was used to determine age at the time of prostate cancer diagnosis. Within the VA dataset, the variables that were high quality and useable in this analysis were limited to PSA, age, and diagnosis of prostate cancer.

### Cancer-Specific Death

Our main outcome variable was prostate cancer death between January 1, 1999 and December 31, 2009. This was the period for which National Death Index (NDI) cause of death data were available. For patients with an indication of death and year of death, the cause of death from prostate cancer was determined using the presence of the ICD-9 code *185* (used before 1999) or the ICD-10 code *C61* (used after 1998). The NDI was previously validated in a chart review by Warren et al. who found a greater than 96% accuracy of the cause of death (21).

### Statistical Analyses

The men in our cohort included those who received no treatment, androgen deprivation alone, radiation alone, surgery, and combination therapies. The study population was categorized into one of the four age groups: 50–59, 60–69, 70–79, and 80–89 years. Men were grouped into 10 PSA ranges at diagnosis: 3–4.9, 5–6.9, 7–9.9, 10–14, 15–19, 20–39, 50–59, 60–99, 100–199, and 200+. For each group, the percentage of men who died of cancer was calculated. The Cochran–Armitage test (22) for trends was used for PSA and for age for various PSA ranges. Cancer-specific death over time was calculated for each combination of age group and PSA range using Kaplan–Meier methods (23). We performed log-rank analysis of differences among PSA ranges for each of the four age groups as well as differences among age groups for each of the 10 PSA ranges. (R version 3.2.1) Multiple hypothesis testing calls for consideration of the conservative family-wise error rate (FWER) or the false discovery rate (FDR). For simplicity, we used the most conservative Bonferroni FWER procedure (24) to adjust the *p*-values for multiple tests. To compare cancer-specific death across the PSA ranges for each of the four age groups, we multiplied each *p*-value by the four tests being performed. To compare cancer-specific death across the age groups for each of the 10 PSA ranges, we multiplied each *p*-value by the 10 tests being performed. Cancer-specific deaths after diagnosis during the 10 years of the study period (1999–2009) were graphed as a function of PSA at diagnosis for each age range separately. Receiver operator characteristic (ROC) curves were created (R version 3.2.1) to determine the discrimination of PSA level and age at diagnosis on prostate cancer death and a simple linear combination of the two (PSA + 1.2 × age) in predicting a higher risk of prostate cancer mortality. On average, we observed that 1.2 years of age had approximately the same influence as 1.0 unit of PSA. For each age group, areas under the curve (AUC) were calculated for PSA level at diagnosis to determine if PSA levels are similarly predictive of prostate cancer death with advancing age.

## Results

### Population Characteristics

Patient characteristics for the 230,081 men aged 50–89 years diagnosed with prostate cancer between 1999 and 2009 are shown in Table 1. For the 10-year study period, mean follow-up was 3.7 years. The mean age at diagnosis was 70.7 years (median 72.2). For the men who died of prostate cancer within the study period, the mean age at diagnosis was 75.5 years (median 77.1). Mean PSA at diagnosis was higher for men aged 70–89 years (mean PSA = 33.3) than men aged 50–69 years (mean PSA = 19.0). Mean and median PSA values at diagnosis were substantially higher for the men who died of prostate cancer, both overall and within each age group. In contrast, mean PSA at death was independent of age among the men who died of prostate cancer (mean PSA = 146.2 with no age trend).

**Table 1:** **Patient demographics and characteristics at diagnosis and cancer death**.

	Age at diagnosis				
Number of men	Overall	50–59	60–69	70–79	80–89
Diagnosed (% of total)	230,081 (100)	32,386 (15)	70,863 (32)	88,051 (36)	38,781 (17)
Died of PCa (% of total)	24,142 (100)	1,371 (6)	4,091 (17)	10,553 (44)	8,127 (34)
**Men diagnosed – prostate cancer**					
Age at diagnosis					
Mean	70.7	56.5	65.2	75.0	83.6
Median (IQR)	72.2 (14.4)	57.5 (4.0)	66.2 (5.1)	74.5 (4.7)	82.5 (3.9)
PSA					
Mean	26.4	19.0	18.9	24.4	51.8
Median (IQR)	5.3 (6.9)	5.2 (5.1)	5.3 (5.2)	5.3 (7.7)	6.2 (13.7)
Follow-up					
Mean	3.7	4.1	3.8	3.8	2.8
Median (IQR)	3.3 (4.2)	3.8 (4.3)	3.3 (4.5)	3.4 (4.1)	2.3 (3.2)
**Men died – prostate cancer**					
Age at diagnosis					
Mean	75.5	56.3	65.8	75.6	83.8
Median (IQR)	77.1 (10.5)	56.8 (4.0)	66.6 (4.7)	76.3 (4.6)	83.1 (4.4)
PSA					
Mean	146.2	218.3	165.0	119.8	159.5
Median (IQR)	12.4 (44.3)	16.4 (84.9)	11.5 (45.6)	10.6 (34.6)	14.9 (51.8)
Follow-up					
Mean	2.8	2.8	3.1	3.1	2.3
Median (IQR)	2.3 (3.2)	2.1 (3.0)	2.5 (3.5)	2.5 (3.4)	1.8 (2.6)

### Cancer-Specific Death Rates

A total of 24,142 (10.5%) of the men in our cohort died of prostate cancer between 1999 and 2009. The numbers of men who were diagnosed with and then died of prostate cancer are shown in Table 2 and are stratified by age group and PSA range. Of men who died of prostate cancer, 54.6% were diagnosed at PSA values of 10 or higher, and 77.4% were diagnosed at age 70 or older. Across all age groups, the percentage of men who died of prostate cancer increased as PSA at diagnosis increased (*p* < 0.0001 for trend). For PSA 0–10, the percentage of men who died of prostate cancer increased with increasing age (*p* < 0.0001 for trend).

**Table 2:** **Distributions of age and PSA at diagnosis for diagnosed men and cancer deaths**.

	Age at diagnosis				
	Overall	50–59	60–69	70–79	80–89
**Number of men diagnosed with prostate cancer vs. PSA at diagnosis**					

**PSA (ng/mL)**					
0–2.9	70,836	8,761	19,112	28,881	14,082
3–4.9	40,912	7,292	15,039	14,200	4,381
5–9.9	67,512	10,729	24,432	25,049	7,302
10–19.9	27,323	3,333	7,419	10,984	5,587
20–49.9	13,217	1,385	2,884	5,094	3,854
50–99.9	4,434	405	840	1,676	1,513
≥100	5,847	481	1,137	2,167	2,062
Total	230,081	32,386	70,863	88,051	38,781

**Number of men died of prostate cancer (% of diagnosed in category)**					

**PSA (ng/mL)**					
0–2.9	4,467 (6)	171 (2)	629 (3)	1,980 (7)	1,687 (12)
3–4.9	2,203 (5)	112 (2)	414 (3)	1,023 (7)	654 (15)
5–9.9	3,301 (6)	263 (2)	855 (3)	2,043 (8)	1,140 (16)
10–19.9	3,749 (14)	181 (5)	618 (8)	1,710 (16)	1,240 (22)
20–49.9	3,553 (27)	196 (14)	545 (19)	1,490 (29)	1,322 (34)
50–99.9	1,933 (44)	128 (32)	306 (36)	805 (48)	694 (46)
≥100	3,936 (67)	320 (67)	724 (64)	1,502 (69)	1,390 (67)
Total	24,142 (10)	1,371 (4)	4,091 (6)	10,553 (12)	8,127 (21)

**Figure 1:**
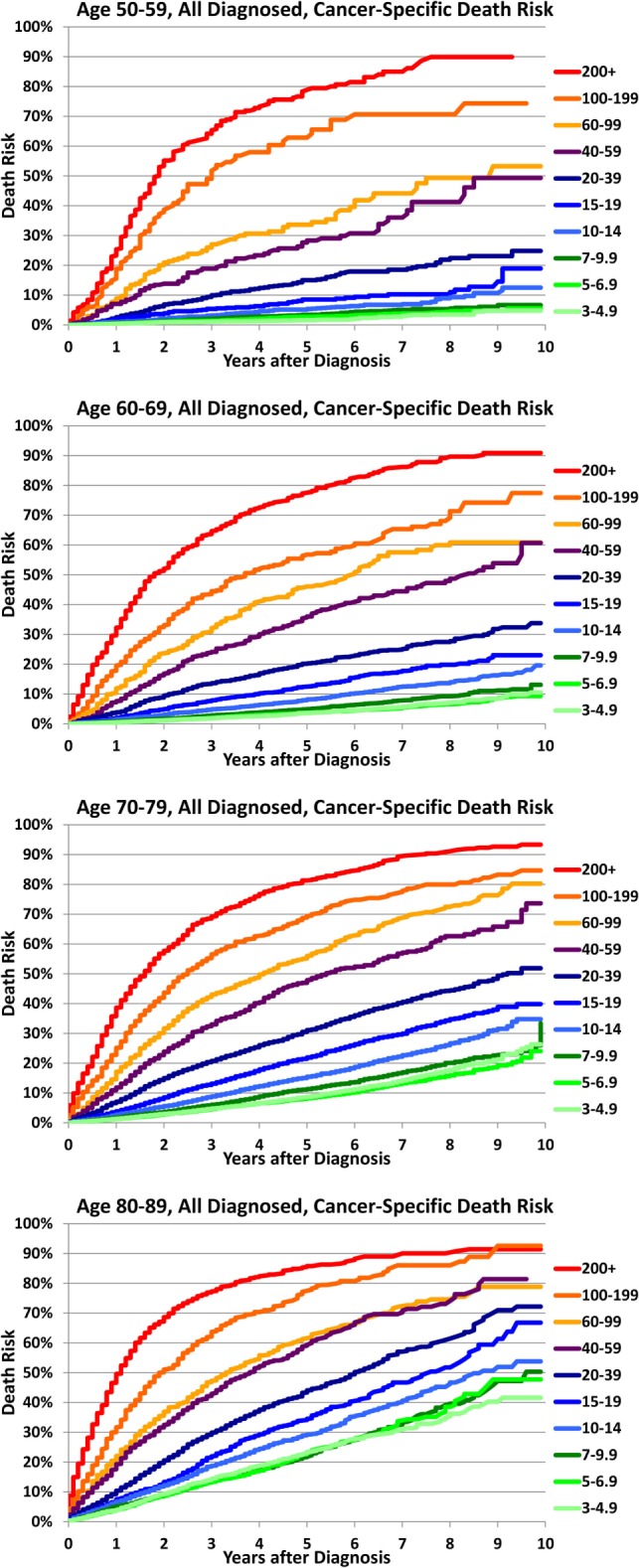
**Cancer-specific death after diagnosis by age and PSA at diagnosis**. For all men aged 50–59 years (a), 60–69 years (b), 70–79 years (c), and 80–89 years (d) diagnosed with prostate cancer, cancer-specific death risk is plotted as a function of time elapsed from diagnosis (years) for various ranges of prostate-specific antigen (PSA) levels at diagnosis.

**Figure 2:**
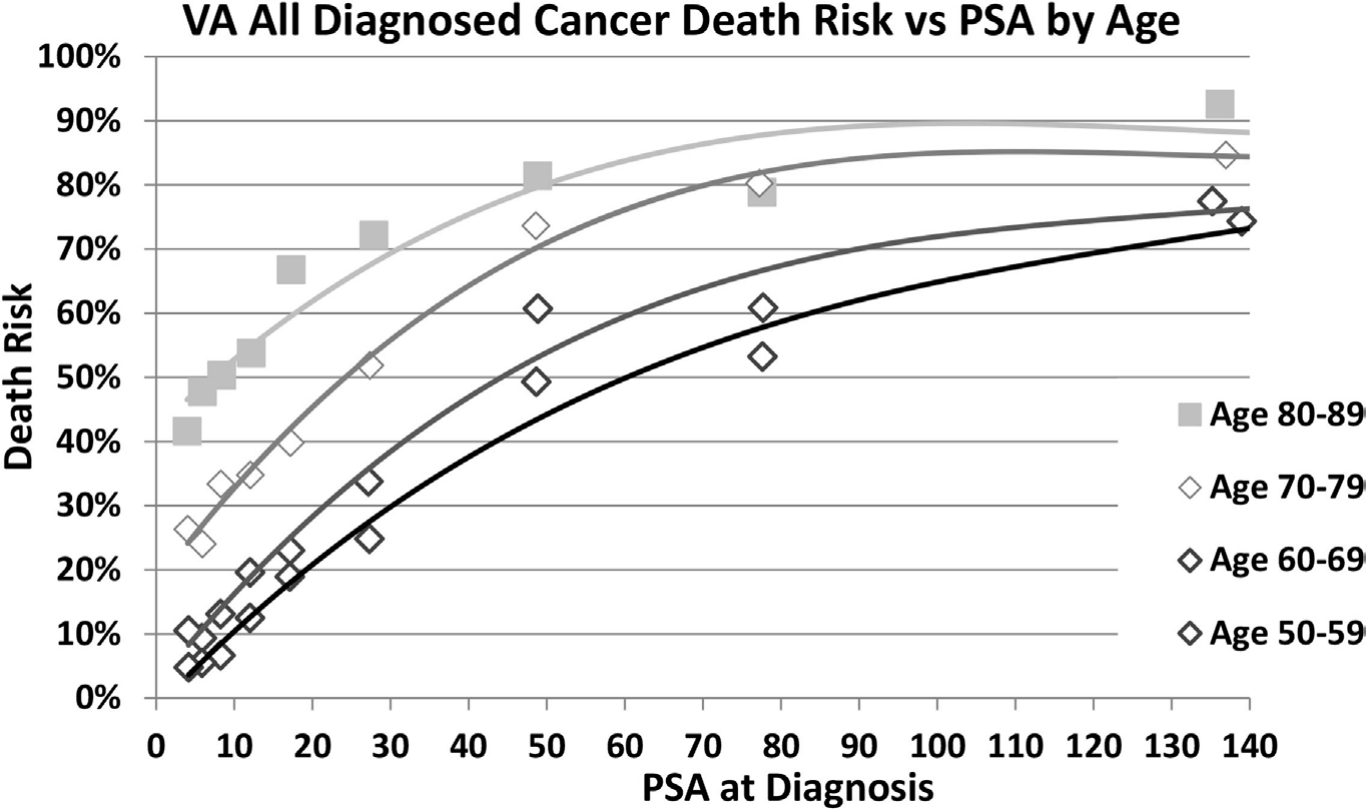
**Cancer-specific death at 10 years by age and PSA at diagnosis**. Death risk from prostate cancer 10 years after diagnosis is plotted as a function of prostate-specific antigen (PSA) level at diagnosis (nanogram per milliliter) for men aged 50–59, 60–69, 70–79, and 80–89 years who were diagnosed with prostate cancer.

Higher PSA values at diagnosis predict a higher risk of cancer-specific death in all age groups as shown in Figure 1. The log-rank test with conservative Bonferroni adjustments to the *p*-values yields *p* < 0.0001 for all four age ranges. In addition, older age groups have an increasing risk of death for the same PSA range with the largest percentage differences at the lowest PSA ranges (test-adjusted log-rank *p* < 0.0001 for all ranges of PSA). In Figure 2, we plotted the actuarial 10-year death rates from Figure 1 as a function of PSA for each age group. The higher cancer-specific death at older ages is seen even at low PSA levels, and this difference in mortality remains at higher levels of PSA.

**Figure 3:**
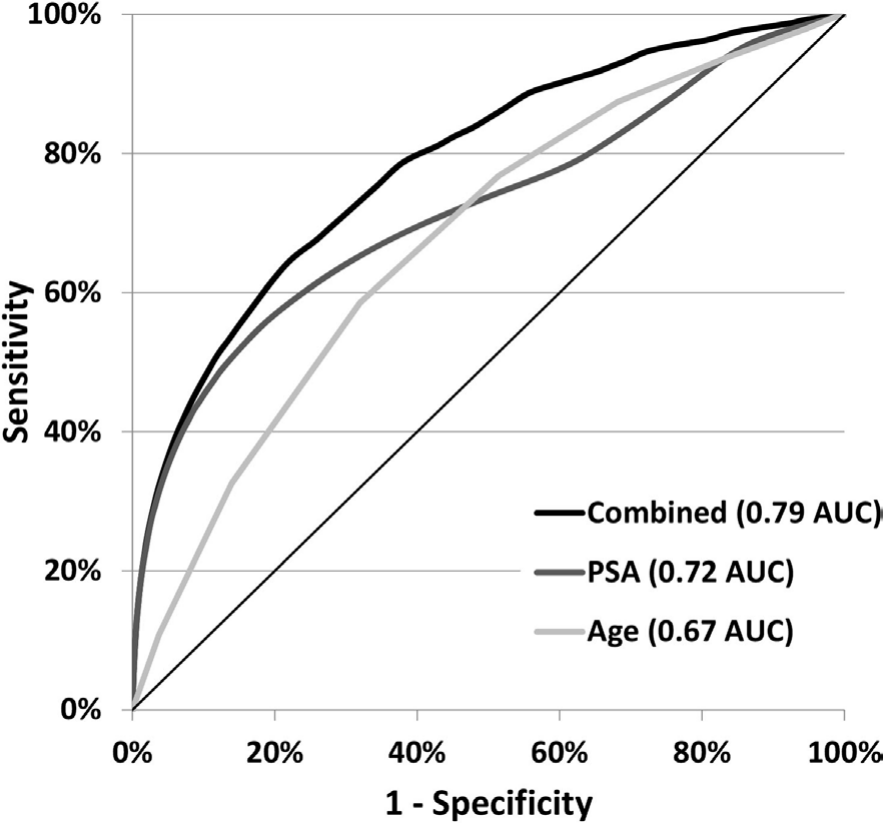
**Cancer-specific death ROC curves for age and PSA at diagnosis**. Receiver operator characteristic (ROC) curves show the sensitivity to prostate cancer death and the specificity to no cancer death for the variables: PSA, age, and a simple linear combination of the two (PSA + 1.2 × age).

PSA and age at diagnosis are strong independent predictors of prostate cancer death compared to no cancer death but are more predictive in combination. The AUCs for the ROC curves were 0.79 for the simple linear function of PSA and age, 0.72 for PSA alone, and 0.67 for age alone, as shown in Figure 3. The AUC for PSA alone was 0.77, 0.73, 0.71, and 0.71 for ages 50–59, 60–69, 70–79, and 80–89 years, respectively.

## Discussion

While many studies, calculators, and nomograms have correlated PSA levels with a higher likelihood of detecting cancer, our study is the first national cohort study to demonstrate a correlation between pre-diagnosis PSA level and 10-year cancer-specific death across increasingly older age groups, even among men 80+.

For example, the PCPT risk calculator estimates the probability that a biopsy will detect cancer for individual men (16–19). For Caucasian men, aged 55 years, without other risk factors, the probability a biopsy will detect cancer increases from 18% at PSA 3.0 ng/mL to 29% at PSA 10.0 ng/mL. For similar men at age 75, the probability increases from 26% at PSA 3.0 ng/mL to 42% at PSA 10.0 ng/mL, which suggests older men might benefit more from a biopsy than younger men. Using the findings of this study, that same older man, if diagnosed with prostate cancer when his PSA is 3, has a 23% chance of dying within 10 years from his prostate cancer, compared to a 33% chance of death if he is not diagnosed until his PSA is 10. While a diagnosis of prostate cancer is important, it is clearly recognized that not all prostate cancers are deadly. This is especially important when we consider screening older men as the increasing risk of cancer diagnosis with age is likely to be offset, to some extent, by reduced life expectancy for older men who may die of other causes before prostate cancer kills them. Utilizing PSA to understand and potentially predict the impact on a man’s survival is even more relevant than predicting the presence of prostate cancer and could significantly inform the decision whether to biopsy older men, especially healthy ones with longer life expectancies.

Of men in this study who died of prostate cancer, 54.6% were diagnosed at high (>10 ng/mL) PSA levels, and 39.0% were diagnosed at very high PSA levels (>20 ng/mL). This suggests that some men were unscreened prior to diagnosis, which is consistent with national screening rates at VA hospitals and clinics (25). It also shows that much of the death risk is associated with PSA levels at diagnosis substantially above the screening guidelines used in the past.

In our study population, 77% of prostate cancer deaths occurred in men diagnosed at age 70–89. This finding suggests that if we are going to reduce prostate cancer death substantially, we should reconsider the management of prostate cancer in older men, especially healthy men with longer life expectancies. The high proportion of all deaths concentrated in older men can be explained, in part, by the increasing risk of death with age for any PSA level. For example, for PSA in the 7–10 range, the cancer-specific death risk 10-years after diagnosis is only 7% for men aged 50–59 years, but 51% for men aged 80–89 years. This substantial upward shift in the risk of death at low PSA levels for older men results in a reduction in the overall increase in cancer death risk as PSA increases and may account for the reduction in the AUC for PSA alone as age increases with AUC of 0.77, 0.73, 0.71, and 0.71 for ages 50–59, 60–69, 70–79, and 80–89 years, respectively.

Our finding that older men have increased cancer-specific mortality risk across all PSA ranges suggests that older men may be presenting with later-stage cancers, have cancers that behave more aggressively, and/or receive less aggressive treatment than younger men. Findings of Tang et al. from the Duke Prostate Center Database (12) and a report by Aizer et al. that included men from the Surveillance, Epidemiology, and End Results Program (SEER) database with a median age of 67 years found older men diagnosed with low-risk prostate cancer had a higher cancer-specific death rate than younger men, whether they were observed or received treatment (26). Similarly, Scosyrev et al. found that older patients who presented with prostate cancer had a higher incidence of metastatic disease at diagnosis (27).

The current dogma that older men do not need treatment because of their age and comorbid conditions may need to give more weight to both their life expectancy and PSA level before diagnosis rather than age alone. For example, the average life expectancy for a 75-year-old man in United States is nearly 12 years to age 87 years. If diagnosed at age 75 with a PSA of 10, our results suggest that his risk of prostate cancer-specific death is likely to exceed 33% at his life expectancy, which may justify aggressive treatment. If his PSA reaches 40, his risk of prostate cancer-specific death is likely to exceed 65%. Clearly, many otherwise healthy older men diagnosed at elevated PSA levels will live long enough to die of prostate cancer with high to very high probabilities and may want to consider treatment options. A more detailed investigation of treatment in older men seems indicated to determine the cause of this high prostate cancer mortality rate and if any intervention could benefit these patients.

Recently identified problems with US National Cancer Institute SEER PSA data reporting (28) have raised questions about other large populations. In contrast to SEER, VA PSA data are a strength of our study because VA PSA values are extracted directly from VA clinical lab databases used for direct patient care.

There are several limitations to this study. It is an observational study that does not control for treatment and other variables. We also cannot determine if the PSA prior to the prostate cancer diagnosis was ordered for screening or diagnostic purpose, and the CDW does not include post-diagnosis prostate cancer clinical stage or grade information to identify men with more advanced disease at a given PSA. Mean follow-up time was 3.7 years, which is short for studies with small populations. In our study, the large group of men analyzed (230,081) allowed estimation of cancer-specific death for the full 10-year study period in spite of the short mean follow-up time.

## Conclusion

PSA remains an important prognostic tool for prostate cancer death even at very advanced ages. Risk of death from prostate cancer directly correlates with the PSA level at diagnosis, and men older than 70 years have an increased risk of death from prostate cancer at all PSA levels, which can help inform decision-making.

The significant cancer-specific mortality rate in the elderly population, even at lower PSA values, suggests further study is needed about ways to decrease prostate cancer mortality in this often-understudied and perhaps underserved population.

## Author Contributions

FM had full access to all of the data in the study and takes responsibility for the integrity of the data and the accuracy of the data analysis. Critical revision of the manuscript for important intellectual content, study concept and design, acquisition, analysis, or interpretation of data: FM, PS, LW, LR, RK, CM, MK, CN, and TN. Drafting of the manuscript: FM, PS, LR, CM, CN, and TN. Statistical analysis: FM, CM, MK, and TN. Obtained funding: LW. Administrative, technical, or material support: FM, PS, LR, CN, and TN. Study supervision: FM, PS, LW, LR, and RK.

## Conflict of Interest Statement

TN is a prostate cancer survivor who wants to help other men make better prostate cancer screening decisions. Toward that end, he founded Soar BioDynamics, Inc., a small start-up company, that he has financed personally with his family. The remaining authors declare that the research was conducted in the absence of any commercial or financial relationships that could be construed as a potential conflict of interest.
